# Cooperation in bioluminescence: understanding the role of autoinducers by a stochastic random resistor model

**DOI:** 10.1140/epje/s10189-023-00352-0

**Published:** 2023-10-09

**Authors:** Eleonora Alfinito, Matteo Beccaria, Maura Cesaria

**Affiliations:** 1 Dipartimento di Matematica e Fisica ‘Ennio De Giorgi’, Università del Salento, Via Arnesano, 73100 Lecce, Italy; 2 Istituto Nazionale di Fisica Nucleare - Sezione di Lecce, Via Arnesano, 73100 Lecce, Italy; 3 National Biodiversity Future Center, 90133 Palermo, Italy

## Abstract

Quorum sensing is a communication mechanism adopted by different bacterial strains for the regulation of gene transcription. It takes place through the exchange of molecules called autoinducers. Bioluminescence is an emergent threshold phenomenon shown by some bacteria strains. Its precise relationship to quorum sensing is a debated topic, particularly regarding the role of the different autoinducers used by bacteria. In this paper, assuming a direct relationship between bioluminescence and quorum sensing, we investigate the role of multiple autoinducers in the bioluminescence response of *Vibrio harveyi*, considered as a model bioluminescent strain, due to its quorum sensing circuitry involving an array of three different autoinducers. Experiments on mutants of this bacterium, obtained by suppression of one or more autoinducers, reveal their relative non-trivial relevance and cooperative interaction patterns. The proposed analysis is implemented on a regular lattice, whose nodes represent microbial entities equipped with *charges*, which represent the ability to up/down regulate the gene expression. Quorum sensing results from a Coulomb-type field, produced by the *charges*. In analogy with random resistor network models, the lattice is permeated by an effective current which accounts for the amount and distribution of the *charges*. We propose that the presence of different autoinducers correspond to a different up/down regulation of gene expression, i.e., to a different way to account for the *charges*. Then, by introducing a modulation of the charge dependence into the current flowing within the network, we show that it is able to describe the bioluminescence exhibited by *V. harveyi* mutants. Furthermore, modulation of the charge dependence allows the interactions between the different autoinducers to be taken into account, providing a prediction regarding the data obtainable under specific growth conditions.

**Graphical abstract:**

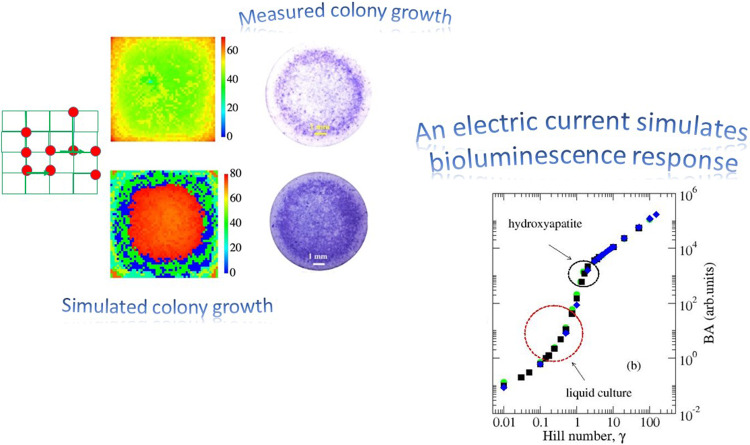

## Introduction

Quorum sensing (QS) describes the cooperative behavior of bacteria in up/down regulation of gene expression. It rules various physiological activities, such as reproduction, biofilm production and development, bioluminescence [1], toxin production, virulence and many others [1, 2]. QS is a long-range phenomenon mediated by certain signaling molecules, called autoinducers (AIs), which are secreted and detected by each bacterium and become effective in driving collective communication only beyond a critical concentration. It is common that bacteria strains communicate through multiple different types of AIs, acting in synergy to implement QS [1, 3, 4]. However, measurements of QS are always indirect and in terms of its effects. Several aspects of this communication mechanism are still to be clarified, such as, for example: (i) the benefits associated with the presence of multiple AIs and their interplay in channeling gene regulation; (ii) to what extent AIs interfere or collaborate and whether this type of association is affected by environmental factors; iii) how different AIs, and the associated isogenic mutants, may modulate different bacterium activities depending on the growth step and involved gene regulation.

Much attention has been devoted to the study of QS in a marine bacterium, *V. harveyi,* also referred to as *V. campbelli.* This bacterium is pathogenic for different marine species and for humans [5], too. *V. harveyi* implements QS by means of three different autoinducers which are produced and detected in parallel: homoserine lactone (denoted HAI-1), the so-called autoinducer-2 (AI-2), and the *V. cholerae* autoinducer-1, termed CAI-1 [1]. The first AI is specific of this *specie*, the second one is an intraspecies signaler, and the last one is specific of the pathogenic bacterium *V. cholerae*, although it plays a main role in the regulation of several activities of *V. harveyi* [1]. Since the bioluminescence yield from *V. harveyi* increases with colony density, bioluminescence is considered an indirect measure of QS [1]. The role of the three AIs in the regulation of bioluminescence was studied in [1] in a liquid culture. The bioluminescence response was analyzed in a set of seven mutants of *V. harveyi* strains, differing in the kind of expressed AIs. In more details, bioluminescence measurements were collected for the so-called wild type (which includes the complete set of three AIs), three mutants, each expressing only one of the AIs, (single-component-mutants), and the three different combinations of mutants expressing two AIs (two-component-mutants). Remarkably, it has been observed that the elimination of one or two AIs drastically reduces the amount of light produced [1], thus signaling that: i) all AIs have an active role in the production of bioluminescence and ii) the latter depends non-linearly on presence of the different autoinducers.Similar results were obtained when growing the bacterium on a solid substrate of interest for clinical and technological applications, namely, hydroxyapatite (HA) [6].

Since the evolutionary dynamics of bacterial colonies is complex and of interest in multiple disciplines, many different models have been developed to describe it. Some of them found on deterministic equations [7–9], other privilege stochastic approaches [10–12], many of them focusing on specific processes regulated by QS, such as biofilm formation [13–15], bioluminescence [8], pattern formation [16, 17], motility [18], and also social behaviors [19].An interesting review of some of the most successful models can be found in [15, 17].

In the present article, we extend a model presented by the authors in [20], based on biological networks [21–23] and introduced to describe the dynamics of colony growth in the case of a single AI. Specifically, in [20],an interplay between a long-range interaction, which represents the ability of AIs to cover long distances, and the possibility for bacteria to reproduce or move over a limited region (nearest neighbors) was proposed. This formulation can be contextualized in the framework of cellular automata modeling for ecological systems [24, 25] with a discrete distribution of cells and discrete/stochastic dynamics of colony growth. The cells are the nodes of a lattice representing the physical space in which the colony develops (*sample-lattice*). Each cell possesses a fictious charge (*c-charge*) which is the source of the long-range interaction and may reproduce/migrate so as to reduce the charge density gradient [20].

Here we perform an investigation regarding bioluminescence as a product of QS in bacteria expressing different types of AIs. Specifically, in the approach of random resistor network models [26], we introduce a quantity similar to an electric current capable of measuring the filling level of the *sample lattice* and the quantity of *c-charges* distributed in it. The electric current depends on the *c-charges *via a Hill-type function [27, 28]. By varying the Hill number, $\gamma$, it is possible to reproduce the results of bioluminescence obtained from different experiments [1, 6] on mutants of *V. harveyi*. In other words, we show that a different bioluminescence response is equivalent to a different evaluation of the *c-charges* in the fictitious current. Finally, the interpretation of $\gamma$ as an expression of cooperation between AIs is discussed.

The paper is organized as follows: The model is described in Section II; the temporal evolution of the colony and its comparison with the experimental data are reported in Section III.1; in Section III.2 the bioluminescence analogue is introduced, the relationship with bioluminescence measurements is proposed, and the cooperativity between AIs is evaluated. The main conclusions are discussed in Section IV.

## Methods

The purpose of this section is to describe the bioluminescence response in bacteria that use multiple autoinducers, as a result of a cooperative alliance between members of a growing colony. Therefore, for the convenience of the reader, we summarize below the colony formation model, previously introduced in [20], then we will present in full details the model novelties bioluminescence.

Colony development is simulated by a network of cells, each of them representing a single bacterium/bacterium aggregate. Each cell is able to communicate with other (alive) cells through a quantity named *c-charge*, *Q*, which represents the effectiveness in terms of genic regulation. Alive cells carry a positive value of the c-charge [20]. The c-charges produce a Coulomb-like field and evolve in such a way to maximize the electrostatic energy of the colony. Both the growth of each c-charge and the colonization of its nearest neighbors are stochastic processes. Furthermore, the exchange of AIs between two cells is mimicked by the activation of a new link. This can happen if there is a potential gradient between the two, with the cell at the higher potential bridging the cell with the lower potential, thus allowing it to increase its charge. Once a connection is opened (link), an increase in AI traffic is described as an increase in the value of the c-charges.

The c-charge are initially randomly distributed on the lattice. Occupancy was set at a relatively small fraction of the grid (5% of the total sites, which is the threshold value to start colony development) with c-charges of unit value.

After this preliminary initialization/inoculation step, the numerical simulation is organized into 3 phases that are repeated at each (discrete) time-step. First we update the c-charge values, then we analyze the associated complex network, and finally we let the colony evolve.

### Charge update

Each cell has a long-range interaction with all the other c-charges of the network by means of the Coulomb-like potential

1 $$V\left({r}{\prime}\right)=\int \frac{\theta \left(r\right)}{\left|r-{r}{\prime}\right|}dr,$$

where $\theta \left(r\right)$ is the charge density and integration is actually discretized by summing over the whole grid. The energy of each c-charge is $e\left({r}^{\mathrm{^{\prime}}}\right)=q\left({r}^{\mathrm{^{\prime}}}\right)V\left({r}^{\mathrm{^{\prime}}}\right)$ and their sum, $\mathrm{energy}\left(t\right)=\int e\left(r\right)dr$ , represents the whole network energy.

Each active cell is able to open a channel (link) with nearest neighboring cells which have lower potential (receivers). This happens with Boltzmann-like probability:

2 $$P\left(r,{r}^{\mathrm{^{\prime}}},t\right)=\mathrm{min}\left(1,\mathrm{exp}\left[-\beta \left(e\left(r\right)-e\left({r}^{\mathrm{^{\prime}}}\right)\right)\right]\right),$$

where:

$${\beta }^{-1}=\frac{1}{2}\sum_{\begin{array}{c}i,j=1\\ (i\ne j)\end{array}}^{N}\frac{Q(i)Q(j)}{\mathrm{Dist}(i,j)}$$ represents the energy of the system at time *t*, i.e. at a certain evolution step (iteration).

When a channel is open, the receiver increases its c-charge according to the rule:

3 $$Q(n)\to Q(n)+\mathrm{floor}\left(\frac{\sigma *\mathrm{links}\left(n\right)}{N}\right)$$

where 1< $\sigma <N$ is a real number whose value determines the efficiency of activation [20] which is comparable with the *basal production rate introduced* in [17]; $\mathrm{links}\left(n\right)$ is the number of sites connected to the *n-th* site. The temporal evolution is discretized, i.e. each time-step corresponds to a single iteration.

### Random resistor network (RRN) analysis

The network is monitored step by step by means of a flow of electrical current. The current, injected from ideal contacts placed on one side of the grid, goes through the active nodes by means of the channels opened in module (i) and is finally collected at the other side. The resistance of these channels varies according to the law:

4 $$R\left(n,m\right)=D\left(n,m\right)[{\rho }_{\mathrm{min}}h\left(n,m\right)+ {\rho }_{\mathrm{Max}} \left(1-h\left(n,m\right)\right)]$$

where ${\rho }_{\mathrm{min}}, {\rho }_{\mathrm{Max}}$ are the minimal/maximal resistivity of the channel [20–23], $D\left(n,m\right)$ is the channel length, i.e. the distance between cells *n* and *m*, and $h$ is the Hill-like function [27, 28]:

5 $$h\left(n,m,t\right)=\frac{{W\left(n,m,t\right)}^{\gamma }}{{g}^{\gamma }+{W\left(n,m,t\right)}^{\gamma }}$$

where $W\left(n,m,t\right)=\frac{Q\left(n,t\right)+Q(m,t)}{4}+\frac{Q\left(n,t-1\right)+Q(m,t-1)}{4}$, and $g, \gamma$ are real numbers. In more details, $\gamma$ , the Hill number, determines how fast the function tends to 1, as $W$ increases, while $g$ gives the $h$ concentration required for 50% output response. The Hill function is a standard tool for the estimation of the affinity of multiple ligands of the same receptor [29]. In that context, the parameter $\gamma$ is a measure of the number of ligand while $g$ is related to the dissociation constant [20, 27].

The present investigation focuses on the cooperation among AIs here introduced by means of the Hill number and experimentally observed in bioluminescence measurements [1, 6]. In experiments it has been noticed that: 1. All the AIs play a role in bioluminescence production; 2. bioluminescence production is due to a specific genic upregulation; 3. The specific contribution of each AI changes with the substrate.

In our modeling, we account for these features by tuning $\gamma$ In fact, tuning $\gamma$ means giving a different weight to the c-charges, which, in the present model, represent the strength of the cells, i.e. their ability to up/down regulated gene expression. Therefore, we propose a law that relates the experimental and simulated data, in particular the fictious current obtained resolving the RRN and the bioluminescence produced by different mutants. In this law, $\gamma$ is decomposed into the different contributions coming from the AIs, calculated for existing data and expected for unavailable data. Specifically: Eqs. (4, 5) describe the sigmoidal trend of the resistance which develops between its maximal (${D\rho }_{\mathrm{max}}$) and minimal values, ${D\rho }_{max}$ and ${D\rho }_{min}$.The total current collected in the out- electrical contact is therefore a measure of the amount of charge in the network and also of its distribution. In agreement with previous investigation [20–23], we use the following values of the parameters of interest: ${\rho }_{\mathrm{min}}=1,$ ${\rho }_{\mathrm{max}}=1000$ in arbitrary units.

The system is solved using the Kirchhoff node law and a standard numerical procedure based on the Gaussian elimination method [20–23].

**Fig. 1:**
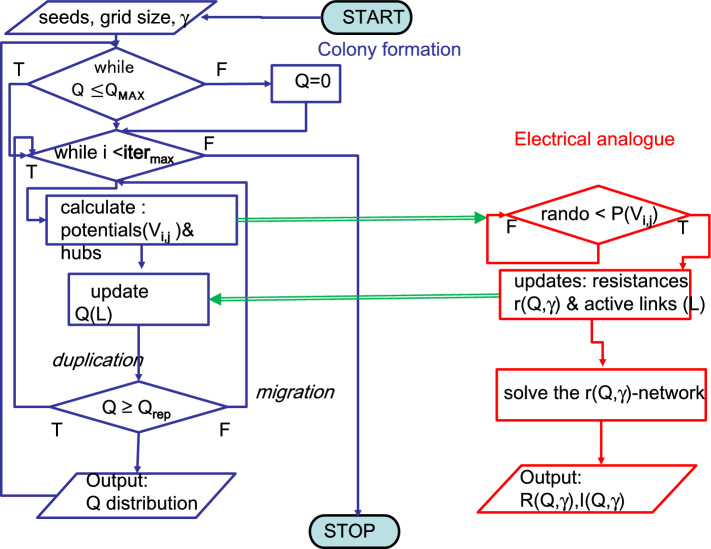
Flow-chart of the procedure. In blue: the colony formation, *Q* indicates the c-charge, Qrep = 2, the reproduction minimal value. In red: the evolution of the current flow inside the network, *r*(*Q*, $\gamma$) is the local resistance, *R*(*Q*, $\gamma$) is the global resistance, I(*Q*, $\gamma$) is the total current

Flow-chart in Fig. 1 (left side) gives a sketch of this procedure.

### Colony spatial evolution

Each cell explores the nearest neighbor nodes and may colonize one of them, transferring half of its value. In the case of a too small charge (*Q* = 1) migration is allowed towards one of the nearest neighbor sites [20]. In both cases (colonization or migration), the sites with the lowest potential are preferentially selected [20], in such a way to easily improve the charge value in the next steps. This choice agrees with the observation that bacteria diffuse on the specific substrate by “sensing” the cell population density [17]. When the c-charge reaches the maximum value, *Q*_*max*_, it is set to ‘dead’ state and is replaced by new c-charges coming from the neighboring sites. In conclusion, the charge value may change according to the possible transitions that we have described:

6 $$\begin{array}{llll} & 0 \to mQ,\quad m \ge 1\\ & nQ \to \frac{n}{2}Q,\quad n \ge 2\\ & {Q_{max}} \to 0.\end{array}$$

These charge dynamics may be considered as a compartmental model [30] which describes birth, reproduction, and death.

In the analysis performed in [20], some aspects concerning the colony and biofilm formation were analyzed in the presence of a single autoinducer. In the present extension, we focus on the role of different auto-inducers regarding the phenomenon of bioluminescence. In particular, we postulate that, at least as far as bioluminescence is concerned, they do not operate independently but may mutually reinforce their efficacy or inhibit each other.

To account for the postulated cooperation among autoinducers, we assume that each of them has a specific cooperativity weight, ${\gamma }_{i}$ and the final value of $\gamma$ in Eq. 5 results by an appropriate combination of them. Specifically, we use a simple “democratic” linear combination:

7 $$\gamma = \sum_{i\in S}{\gamma }_{i}, $$

where *S* is the set of AIs present in each experiment.

The flow-chart of the procedure is illustrated in Fig. 1.

In conclusion, the model of QS and bioluminescence we have proposed is based on the interplay between a long-range interaction among c-charges, introduced to account for the potentially unlimited diffusion of AIs in real samples, and the short-range dynamics of cell proliferation. The specific choice of a Coulomb-like ${1}/{r}^{2}.$ interaction is a simple possibility. Although the electrical interaction in our model is a fictitious one, a Coulomb law is nonetheless inspired by the existence of electrical bacteria which interact among them and with environment by exchanging electrical charges [31, 32]. Due to its dual dynamics, our model is beyond the class *lattice-based interacting random-walk models* [33]. The c-charge dynamics induces some notable differences with respect to the model based on interacting or non-interacting lattices. In particular, the stationary state is not static but is due to continuous death-birth processes; *Q* > 1 can be interpreted as a crowding effect; the distribution of c-charges changes continuously in a coordinated way, and gives rise to spherical wave-like patterns [34], as will be seen in the next section.

## Simulation results

A set of preliminary tests was performed to calibrate and tune the input parameters of the model parameters in such a way to capture some general behaviors shown by the bacterium strain under study. All simulations were performed on a 50 × 50 grid.

### Colony evolution

**Fig. 2:**
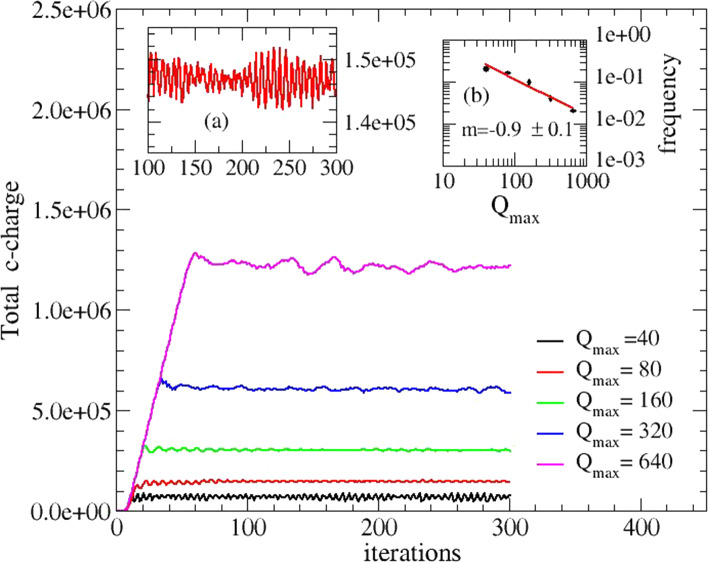
Time evolution of the total c-charge in the network. The charge has been calculated using different values of the *Q*_max_. Each *Q*_max_ value produces a different frequency of oscillation. A zoom of the c-charge oscillations (*Q*_max_ = 80) is reported in the inset (**a**); in the inset (**b**) the mean value of the frequency is reported vs. *Q*_max_ and a power law is detected with exponent m = -0.9. Each iteration step corresponds to a unspecified time interval

Here, we analyzed the effects of different values of *Q*_max_, a parameter linked to the cell maximal power, and, in turn, to its lifetime. The value of *Q*_max_ has been varied over a wide interval (see Fig. 2). In all cases, the network shows a coordinated response, with the following three features:

(i) A stationary state is reached in which the total charge of the network reaches its asymptotic value which linearly depends on *Q*_max_ (Fig. 2-main). The onset of the asymptotic behavior also depends linearly on* Q*_max_.
(ii) Oscillations around the asymptotic value appear, with constant frequency and amplitude modulation (Fig. 2, panel a).
(iii) The oscillation frequency with respect to the *Q*_max_ value is sublinear (Fig. 2, panel b).

From the simulations, we conclude that the choice of a specific lifetime value does not affect the general dynamics of the system. On the other side, we notice the onset of collective oscillations, a phenomenon present in various ecological systems [32]; here it is associated to the lifetime value: longer lifetimes allow the cells to become stronger and more stable ( slower frequency). The existence of characteristic coordinate oscillations in bacteria is the subject of much experimental and theoretical research [35–38] and has been ascribed to different mechanisms, like QS itself, which can induce circadian rhythms [38], to a spontaneous chiral symmetry breaking [35], or to selected combinations of growth-rate in competing bacteria strains [36], although an explicit dependence of collective oscillations on cell lifetime is a topic which deserves further investigations [37].

**Fig. 3:**
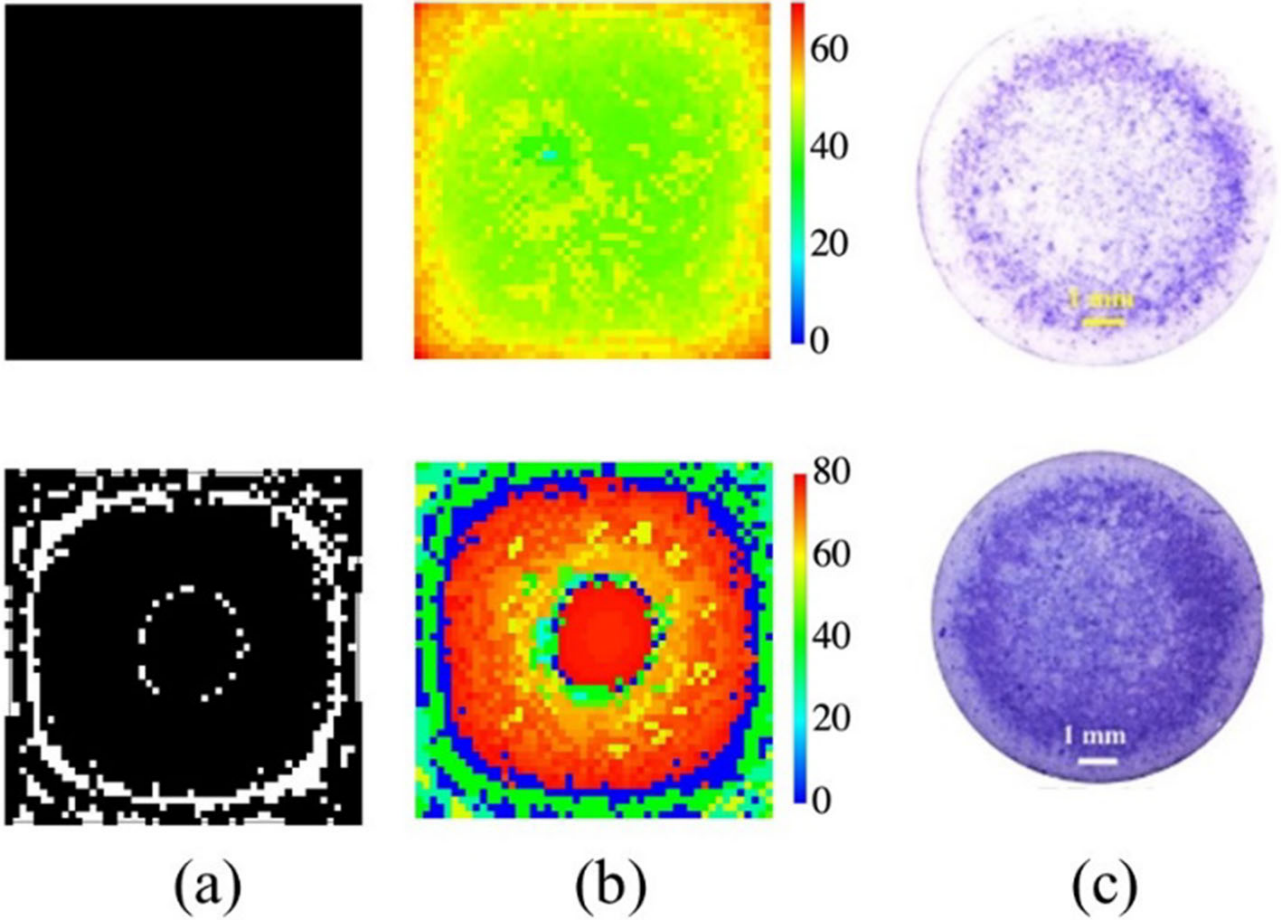
Comparison of simulated and experimental colony growth. **a** Simulation of the network-cells at iteration 15 and 25 (top, bottom) black pixel means occupied site; **b** Simulation of the c-charges at iterations 15 and 25 (top, bottom), color scale from blue to red; **c** Images acquired by stereomicroscopy of mature biofilms grown for 24 and 48 h (top, bottom) onto hydroxyapatite substrates of the wild type strain BB120 [6]

A typical evolution of the spatial distribution of cells and their charges is shown in Fig. 3a. The empty and occupied sites (cells) at iteration 15 and 25 are reported, as well as the amount of c-charge they possess, Fig. 3b. Finally, Fig. 3c, the simulations are compared with the evolution of the bacterium *V. harveyi* (wild type), grown on HA [6] and a general agreement can be observed. In particular, in both simulations and experiments, the formation of circular patterns is observed, which is a non-trivial feature of the growth. Indeed, this type of patterns has been observed quite frequently in several bacteria strains [39, 40] and its origin is supposed to be linked to various factors, such as the characteristics of the substrate [41], the concentration of nutrients [42], crowding, and also to alternate phases of migration and consolidation [17, 43].

In particular, in the present model, cell diffusion and charge growth are driven by a gradient of charge density (Eqs.1, 2) which induces some spatial heterogeneities of the simulated configurations, as observed in [17]. On the other hand, the patterns observed in colonies grown on hydroxyapatite (Fig. 3c), could derive from the finite dimensions of the substrate [41], even if it is not possible to exclude other factors linked to the growth dynamics of the specific strain. Finally, the very mechanism of colony diffusion on real samples is probably a complex interplay between biochemical and physical/space factors [42].

### Bioluminescence-analogue (BA)

**Fig. 4:**
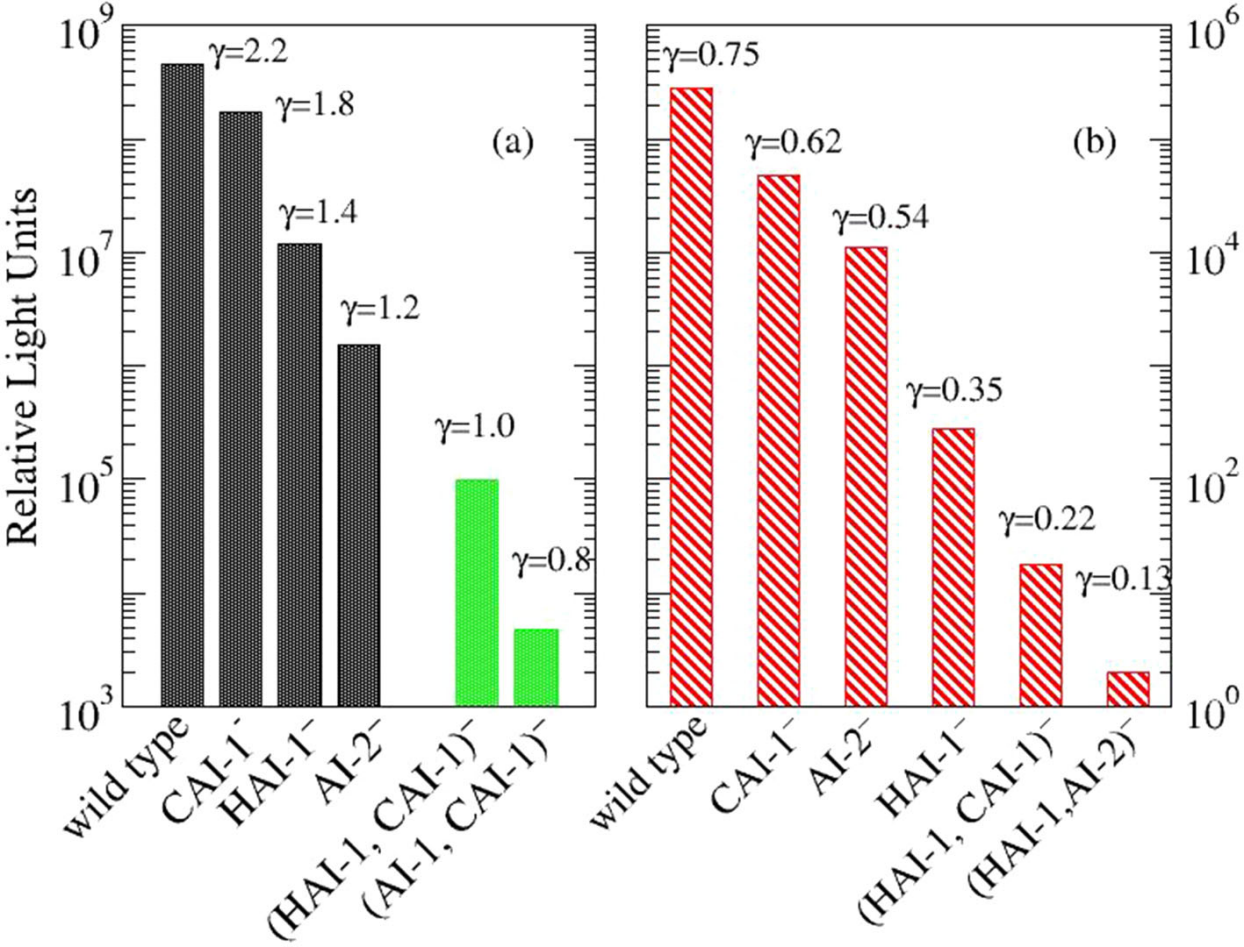
Bioluminescence data as reported in: [6] (**a**) and [1] (**b**). The Hill number, $\gamma$, is due to the collaboration of all the AIs contributing in the specific mutant and is described in Eq. (7). The green bars in panel (**a**) are simulated by using Eqs. (7, 8)

Measurements of bioluminescence ( BL) performed on different mutants of *V. harveyi* are reported in [1] and also in the more recent investigation [6].The wild-type strain of this bacterium communicates through 3 different autoinducers, while mutants are obtained by depriving the strain of one or two of them. Both papers [1, 6] report that the wild type produces the highest luminescence. Some differences appear in the role of different mutants in investigations performed in liquid culture [1] or on HA [6] (see Fig. 4). In particular, in a liquid medium, bioluminescence due to mutants deprived of a single AI (AI^−^) is reduced in the following order [1]: CAI-1^−^ > AI-2^−^ > HAI-1^−^. Single- component mutants are dim or dark [1].

The two-component mutants show a reduction in light of about 83%, 96% and 99%, respectively. This shows that bioluminescence is effective only in the wild type and, furthermore, assigns to CAI-1 a marginal role in its production. On the other hand, although marginal, CAI-1 is necessary to obtain the maximal response (wild type). Mutants deprived of HAI-1 give a poor response in terms of bioluminescence although better than the one given by mutant deprived of HAI-1 and also of CAI-1. On the other hand, for growth on hydroxyapatite substrate [6], bioluminescence emission from the two-component mutants follows the order: CAI-1^-^ > HAI-1^-^ > AI-2^-^ and single-component mutants have not been analyzed. In colonies grown on HA, CAI-1 has a minor role compared to that observed in liquid culture and the reduction of bioluminescence is about 60%, while deprivation of HAI-1 reduces the signal by about 96% and that of AI-2 by 99%, thus shifting the role of dominant auto-inducer from HAI-1 to AI-2.

This rather complex interaction between the three AIs has not been the subject of previous theoretical investigations. Our Ansatz is that there is a correspondence between the physical BL signal and the fictitious electric current coming from Eqs. (4, 5).

**Fig. 5:**
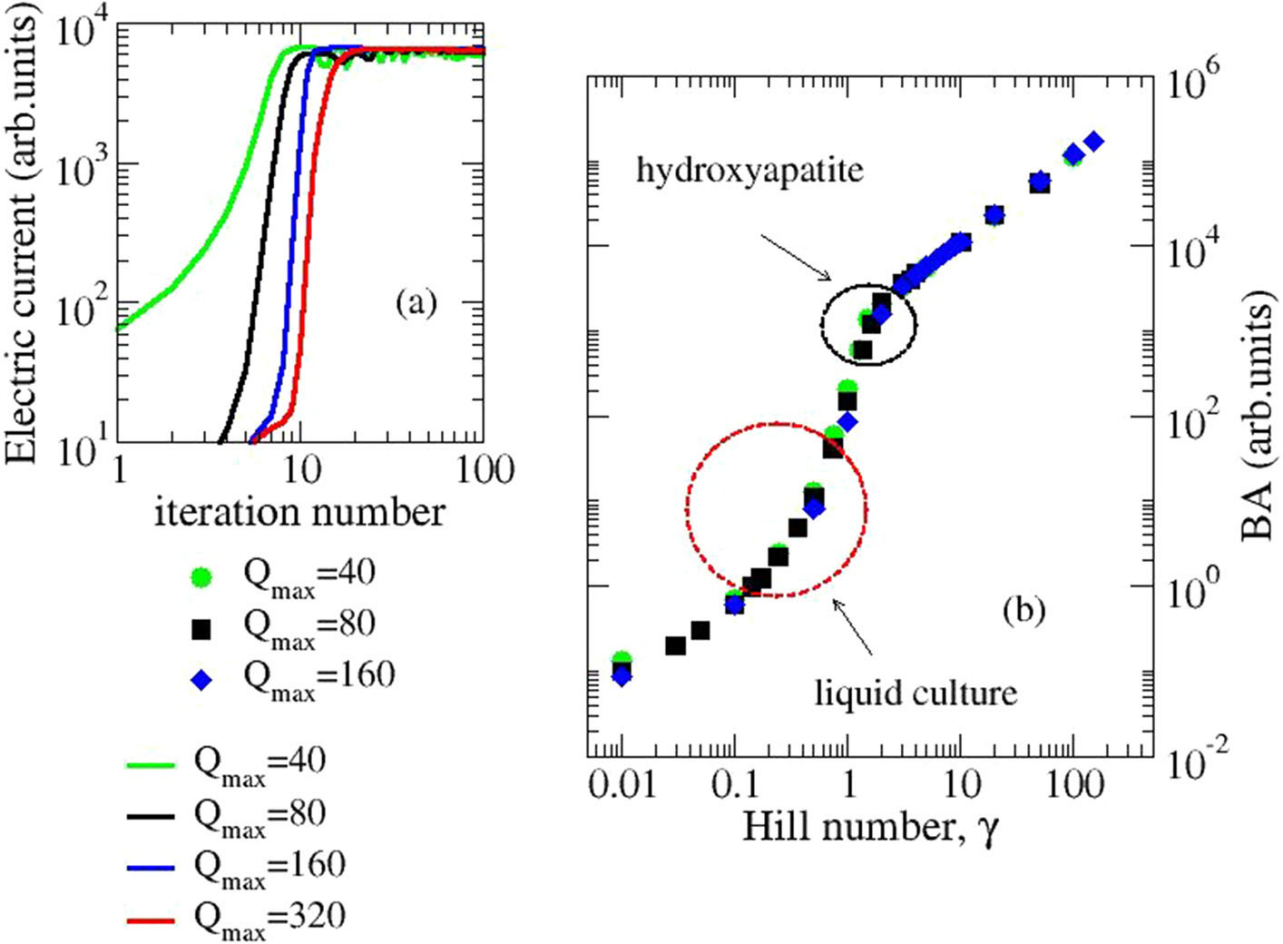
Simulated electric current. **a** Evolution in time for different value of Q_max_, and assigned Hill number ($\gamma$ = 5); **b** Asymptotic value (BA) calculated for different values of the Hill number. The ellipses highlight the curve regions corresponding to experimental data ( see also Fig. 4)

The time evolution of the fictitious current (Fig. 5a), calculated for a fixed $\gamma$ value, has been indicated in [20] as a useful tool for monitoring network connectivity. On the other hand, BL occurs when the colony has exceeded a critical size [1], so we analyze the fictitious electric current in its asymptotic behavior, which we will henceforth call the bioluminescence analogue, $BA(\gamma )$ and we will analyze it as $\gamma$ varies (Fig. 5b): it depends little on *Q*_*max*_ and has a characteristic sigmoidal shape, due to the Hill function. We notice that using different values ​​of $\gamma$ means attributing a different weight to the c-charges present in the network (Eq. 5).

We will assume that this could tell us something more about the mechanisms of cooperation within the colony-equivalent. In particular, we can imagine situations in which individuals with different aptitudes for cooperation respond differently to similar conditions of space occupation and colony evolution.

In order to compare the model with the above mentioned measurements, one has to assume a specific relation between the stochastic current $BA(\gamma )$ and the bioluminescence BL. We considered a simple power-law relation:

8 $$BL=k {BA}^{a}$$

where $k$ is a normalization constant and α > 0 a fixed exponent.

We simultaneously fitted $k$, *α*, and the three cooperative weights using the function $BA(\gamma )$ with Q_max_ = 80 (Fig. 5b). Remarkably, despite the non-linearity of the dependence $BA=BA(\gamma )$ we could obtain a very good agreement with the experimental data reported in [1, 6] (see Fig. 4). This is non-trivial because $BA(\gamma )$ saturates at large values of $\gamma$ (see Fig. 4) and the non-linear fit may have simply no real solution. Our best fit parameters are collected in Table 1 and the final value of $\gamma$, for each experimental data is reported in Fig. 4.

**Table 1:** Fitting data used to reproduce experiments with simulations based on Eqs. 5–8. The exponent *α* in Eq. (8) is 3.0 for both the experiments ([1], liquid culture, [6], HA) and *k* is 2.6 for liquid culture and 0.03 for HA

Autoinducer	*γ*_i_ -liquid culture	*γ*_i_ -HA
HAI-1	0.40	0.81
AI-2	0.22	1.0
CAI-1	0.13	0.37

Finally, by using the Eqs. (7, 8) we can predict the bioluminescence from single-component mutants. In Fig. 4, green bars describe the expected values for mutants deprived of HAI-1 and CAI-1, and mutants deprived of AI-2 and CAI-1. Data concerning mutants deprived of HAI-1 and AI-2 have been not reported because too small.

In conclusion, using the simple power law of Eq. (8), for each of the two experimental setups (liquid environment or solid substrate), the measured data belong to the same ideal curve of Fig. 5b, which reports the asymptotic current value, i.e. obtained when the lattice is filled and mediated over several processes of death-birth. This is obtained simply cutting off the non-stationary initial stage of evolution. Each point on that curve derives from the cooperation of the AIs present in the mutant, as expressed in Eq. (7). The specific cooperative weight of each AI does not change with the mutant, i.e. wild-type, single and two-component mutant, but depends on the type of substrate.

## Conclusions and discussion

The phenomenology of bacteria colonies and, more generally, of active matter systems, is diversified and of interest to multiple disciplines, from ecology to medicine, from physics to the social sciences. In fact, bacteria constitute ecosystems with some general rules and multiple exceptions. This explains the plethora of existing models that use different techniques to capture at least some general phenomena, from motility to colonization, enemy counteraction, drug resistance and predation strategies, in some of their specific manifestations.

A fascinating aspect of this subject is the role of collective behavior over that of the individual. The behavior of the individual, however complex, always aims at improving the community wellbeing. Often, this happens by means of a coordinate communication mechanism named quorum sensing (QS), which is made possible by diffusion and successive capture (autocrine signaling) of small molecules (AIs) all around the environment.

The ramification of research activities in this field is rather complex concerning methods (deterministic or stochastic), strategies (agent-based, mean field, discrete), structures (lattice-based, lattice-free), and finally aims (mainly biofilm formation, emerging features). It is also of interest for gaming and social sciences (sociomicrobiology).

In the present paper, we have proposed a model for the interpretation of bioluminescence measurements in mutants of a bacterium, *V. harveyi*, which uses a QS system based on three different AIs. A few bacteria strains are bioluminescent, i.e. their colony formation goes with light emission, bioluminescence, which is an emergent phenomenon and, in some cases reaches enormous dimensions, so as to be visible even from satellites [44].

This model upgrades a previous investigation by the authors in which a single AI- QS was described as the result an action at a distance instead of the exchange of physical objects. In this framework, the system evolves to maximize its (fictitious) electrostatic energy and, in the process, it promotes the formation of a bacteria colony. In terms of real systems, this could happen extracting energy/food from the environment [45], thus allowing the system to reach a maximal energy state; the availability of food is considered unlimited.

In the present description, the colony evolution is monitored by an auxiliary fictitious electric current which depends in a non-linear way, according to a Hill function, on the power and the distribution of bacteria. The asymptotic value of this current is here correlated with the observed bioluminescence.

A mixed population of cooperative and non-cooperative autoinducers, AIs, competing for the public goods seems to underlie the different manifestations of QS [46, 47]. As an example, bioluminescence in *V. harveyi* is mainly sustained by two of the three AIs, i.e., HAI-1 and AI-2, with CAI-1 being a cheater in this respect [1, 6]. This observation could be translated into terms of cooperative coefficients for each of the AIs. Each (numerical) experiment is characterized by a single Hill exponent and the problem is how to combine the single AIs coefficient in a universal function able to reproduce all the configurations (one, two or all AIs), corresponding to the different kinds of mutants. Our analysis shows that data are well reproduced by employing the simple linear combination in Eq. 7 combined with the phenomenological exponent *α* = 3, relating the observed bioluminescence and the stochastic current of the model.

In agreement with experiments [1], the cooperative weights of the AIs turn out to be in the order: HAI-1 > AI-2 > CAI-1 ( see Table 1).The same fitting formula (8) describes the bioluminescence of *V. harveyi* grown on hydroxyapatite [6], reproducing in this case the cooperative order: AI-2 > HAI-1 > CAI-1. Finally, using the calculated cooperative weights we can predict the BL of single-component mutants of *V. harveyi* grown on hydroxyapatite. Data are reported in Fig. 4.

The question concerning the specific role of each AIs in different manifestations of QS still remains open. In line with the source-sink energy dynamics postulated in [48] for the transfer of energy from bioluminescence to other features, mutants containing CAI-1 probably exploit energy for activities of the colony different from luminescence production. The opposite behavior is observed in HAI-1 and AI-2 and therefore switching on/off the genes that produce these AIs should help the colony to face different environmental conditions, i.e. the amount of available food.

The model here proposed is inspired by many previous speculations, in particular concerning the spreading on a limited lattice, the limited lifetime of each cell, the short range dynamics of cell proliferation and diffusion as well as the cellular automata and random resistor networks [15, 17, 24, 25, 33]. On the other hand, it presents some major novelties concerning: *a.* The introduction of a long-range interaction among cells, mediated by a specific property of the cells called c-charge. This interaction, in turn, leads to a positive feedback loop in which the smaller charges are made to grow by the higher ones; *b.* A short-range oriented mechanism of cell duplication, which naturally introduces heterogeneities in the c-charges distribution; *c.* A random resistor network description of the evolving network, in which the current permeating the lattice depends on the c-charge distribution and is used to evaluate the associated bioluminescence by using a power law relationship. *d.* the concept that how the value of the c-charges is evaluated in the calculation of the effective current is a measure of how well each AI is able to perform gene regulation.

As a final remark and as an open issue left for future work, the model discussed in this paper still does not take into account the specific role of each AIs in the colony development (its growth rate and shape fine structure). In our analysis, we only modelized the role of three different AIs in producing bioluminescence. This kind of decoupling is motivated by the remark that BL data [1, 6] concern only well-consolidated colonies, i.e. they are not dynamical data. The introduction of different AIs in the colony-growth dynamics should involve some of the model parameters, useful for accounting, for example, to describe different rates of growth [6, 48]. That is the case of *Q*_max_ whose modulation strongly affects the amount of the network c-charges (Fig. 2). On the other hand, *Q*_max_ does not explicitly affect the values of specific cooperative weights (Eq. 8).

Finally, the present study supports the idea that the interaction among multiple AIs sustains the fitness of the colony, helping it to regulate growth and other physiological functions in the presence of different environmental conditions [45].

## Data Availability

Data will be made available on reasonable request.
